# Release of Chromium from Orthopaedic Arthroplasties

**DOI:** 10.2174/1874325000802010010

**Published:** 2008-01-24

**Authors:** G.A. Afolaranmi, J Tettey, R.M.D Meek, M.H Grant

**Affiliations:** 1 Strathclyde Institute of Pharmacy and Biomedical Sciences; 2 Department of Orthopaedic Surgery, Southern General Hospital, Glasgow, UK; 3 Bioengineering Unit, University of Strathclyde, UK

## Abstract

Many orthopaedic implants are composed of alloys containing chromium. Of particular relevance is the increasing number of Cobalt Chromium bearing arthroplasies being inserted into young patients with osteoarthritis. Such implants will release chromium ions. These patients will be exposed to the released chromium for over 50 years in some cases. The subsequent chromium ion metabolism and redistribution in fluid and tissue compartments is complex. In addition, the potential biological effects of chromium are also controversial, including DNA and chromosomal damage, reduction in CD8 lymphocyte levels and possible hypersensitivity reactions (ALVAL). The establishment of these issues and the measurement of chromium in biological fluids is the subject of this review.

## INTRODUCTION

Orthopaedic arthroplasties are artificial devices which are used to replace damaged joints in the body, e.g. hip, knee, finger and shoulder. They were initially mainly used by the elderly but, are now used in increasing numbers across the populace. About 0.16-0.2% of population per year in industrial countries undergo total hip joint arthroplasty and the recipients are from all age groups ranging from 20-80 years of age. More than 50,000 total hip replacements (THRs) are implanted in the United Kingdom per year [1].

## BIOMATERIALS IN ORTHOPAEDIC IMPLANTS

Biomaterials are materials which are either natural or man made and which are used to aid or totally replace the functions of living tissues. A bone implant should have an identical loading response to natural bone. The average load on a hip bone has been estimated to be thrice the body weight and this may increase to as high as ten times the body weight during strenuous exercise. An ideal bone implant should therefore exhibit good and appropriate mechanical properties identical to natural bones and be highly biocompatible with existing tissues [2].

Various biomaterials have been used in the design of the elements of THR. Polymeric materials, as a result of their mechanical weakness, have been considered unsuitable for meeting the stress deformation requirements in THR components while ceramics are biocompatible with existing tissues but are brittle and designs must take this into account. Metals have good mechanical properties, but poor biocompatibility properties, cause stress shielding and a systemic release of ions [2].

A hip arthroplasty in most cases is made up of a metallic or ceramic component articulating with a metal, ceramic or polyethylene surface [3]. The combinations possible include metal [stainless steel or cobalt Chromium (CoCr)) on-ultra high molecular weight polyethylene (UHMWPE)], metal-on-metal (CoCr), ceramic on polyethylene, ceramic on ceramic or more recently ceramic on CoCr [4,5].

Many types of metal-on-metal (M/M) hip implants were developed and implanted in significant numbers in the 1960s. They were however mainly replaced in the UK by the metal on polyethylene implants in the mid 1970s as a result of early reports of seizing and loosening and the success of the Charnley and Exeter hip arthroplasties. The seizing and loosening of M/M arthroplasties was associated with metal staining due to wear and corrosion of the metal-on-metal articulating bearing surfaces [6]. However, the success of the metal on polyethylene implant has been tempered in more active patients as UHMWPE wear debris generated at its articulating surfaces is believed to play an important role in periprosthetic osteolysis which leads ultimately to aseptic loosening. In genetically susceptible patients inflammation and bone resorption occurs at the implant-bone interface as a result of the release of cytokines for example, (interleukin 1β, interleukin-6 and tumour necrosis factor α) caused by an active phagocytosis of the generated UHMWPE wear particles by macrophages [7]. Metal-on-metal total hip replacement (M/M THRs) staged a comeback in the 1990s when THRs made from cobalt-chromium alloy were introduced to overcome the problem of polyethylene wear induced osteolysis. The reintroduction was based on good long term results associated with Mckee-Farrar THRs (M/M coupling used in 1960s) which achieved excellent results even at 20 years follow up [6,8-10].

The modern M/M prostheses are slightly different in design from the 1960s M/M. The latter were crude in design while the modern day M/M implants have a great range of sizes and are finished with a greater accuracy. Extremely low linear and volumetric wear rates have been reported [1,5,10-12] and osteolysis as seen with polyethylene particles is considered to be relatively rare in M/M joints. Extremely low linear wear rate of 4-12µm/year in M/M implants compared with a linear wear rate of 100-300µm/year in polyethylene cups have been reported [12]. Doorn and colleagues reported that metal wear particles from M/M implants were of smaller diameter (<0.1µm) compared to polyethylene wear particles (0.5µm) from conventional implants [11]. Green and coworkers reported that only polyethylene particles of size 0.3-10µm induced the release of cytokines by macrophages with subsequent osteolysis [7].

After a rapid wear rate for the first year after implantation, as a result of an initial conditioning phase, most M/M arthroplasties have a constant low wear rate [12]. This is considered particularly important for young and physically active patients who otherwise may have to undergo several revision surgeries in their lifetime [13].

## METAL ORTHOPAEDIC IMPLANTS

Stainless steel, cobalt-chromium and titanium alloys are used in various orthopaedic implants. The compositions of the main alloys used, are outlined by the International Organisation for Standardisation, published by British Standards [14] and shown in Table **1**.

## METAL IONS FROM ORTHOPAEDIC IMPLANTS

Corrosion is an electrochemical reaction which all metals in contact with biological systems undergo. It leads to the formation of metal ions and these metal ions may trigger hypersensitivity reactions and affect the immune response system.

Metal ions and debris have been shown to be released from orthopedic implants which are made of stainless steel and cobalt -chrome alloys [11,16,17]. Chromium, molybdenum, silicon, iron, and manganese are the ions released from stainless steel implants while titanium, aluminium, vanadium, and niobium are released from implants that are made of titanium alloys [3]. This in-situ degradation of an implant is undesirable since it decreases the structural integrity of the implant and also releases products which may elicit an adverse biological reaction in the host [18].

Biological risks associated with the released metal ions have been identified to include those from wear debris, colloidal organometallic complexes, free metal ions and inorganic metal salts or oxides formed [19]. Theoretically, high ion levels have been implicated in delayed-type hypersensitivity, organ toxicity and carcinogenesis and are associated with dermatitis, urticaria and vasculitis. The presence of a joint replacement device, especially a failed metal implant, has been shown to predispose patients to dermal sensitivity when compared with the general population [15]. Evans and coworkers in 1974 [20] identified metal sensitivity as a cause of bone necrosis and implant failure but it remains unclear either metal sensitivity occurs in patients as a result of implant failure or the implant failed because of a preexisting metal sensitivity in the patients [15, 21]. Aseptic lymphocytic vasculitic associated lesions (ALVAL) which may represent immunological response to metal wear debris [22,23] has been documented in tissues around M/M implants, this lymphocytic infiltration was absent in control group of tissues obtained at revisions of implants without cobalt, chromium and nickel [22] and in metal-on-polyethylene implants [24]. Cells and tissues around and distant from the implant are exposed to the toxic effects of the released metal ions and wear debris. Areas of tissue necrosis with associated visible metal particles have been identified from histological studies of tissues retrieved from revised metal prostheses [11,16]. Metal ion concentrations have been measured in patients with implants. Elevated metal ion concentrations in serum [9, 13,25-27], erythrocytes [19, 28], urine [9, 26, 28], whole blood [29-31], tissue [32, 33] and organs [34] have all been reported. Merritt & Rodrigo in 1996 observed that longer implant time *in situ* is associated with increased implant degradation products which would theoretically predispose to an increased metal sensitivity [35].

The published reports on the modern, second-generation M/M bearings to date have uniformly shown substantial elevations in serum, blood, erythrocyte, and/or urine Co and Cr ion levels in comparison with control patients without implants and/or in comparison with patients with metal-on-polyethylene bearings [28, 36]. It has been confirmed that the increased ion level is due to the presence of a M/M implant since ion levels showed a decline following the removal of the M/M implant [13]. The migration of these metal ions and wear particles to distant sites is of potential concern, considering the highly toxic and carcinogenic nature of these metal ions. Of particular relevance to M/M CoCr hip arthroplasties is the Chromium (Cr) ion. Several investigators have measured the concentrations of Cr ions following hip replacement. These findings including the levels of ions in normal healthy controls are summarized in Table **2**.

## CHROMIUM

Chromium, the 24th element in the periodic table, is mainly used as an alloying element in steels where it contributes to hardness, tempering and resistance to oxidation. It is naturally occurring and is found in soil, plants and animals. It occurs in various valences, including chromium (0), chromium (III) and chromium (VI). Chromium (III), which is the most stable form, occurs naturally in the environment whereas industrial processes produce chromium (0) and chromium (VI). Chromium occurs primarily as chromium (III) or as chromium (VI) which is a strong oxidizing agent.

The form of chromium found in foods is Cr (III) and it is essential for maintaining normal glucose tolerance factor and metabolism. Its deficiency in humans will lead to impaired glucose tolerance, glycosuria, fasting hyperglycemia, and elevated circulating insulin and glucagon [43]. Bran breakfast cereals, broccoli, green beans and some brands of beer and wine are all high in trivalent chromium [44]. Trivalent chromium has a large safety range and no documented signs of its toxicity in any of the nutritional studies at levels up to 1mg per day have been reported [43]. However, Cr (VI) is a strong oxidizing agent, is toxic and crosses cell membranes. Cr (VI) is classified by WHO as a class 1 human carcinogen [45].

## DISTRIBUTION OF CHROMIUM IN BLOOD

**Fig. (1):**
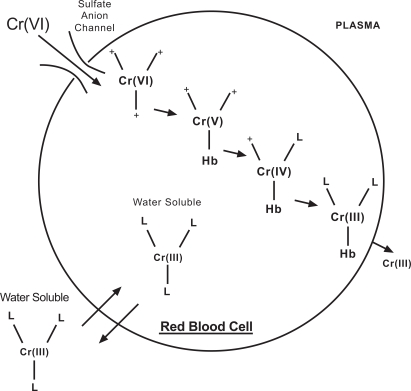
Cr (VI) and Cr (III) uptake in red blood cells adapted from [54]. This schematic depicts how Cr (VI) enters the RBC readily, while chromium (III) on the other hand moves across the cell membrane via much slower diffusion and through other processes related to the chemical structure of the attached ligands [54].

Hexavalent chromium compounds that enter the bloodstream are actively transported into red blood cells (RBC) *via *non specific anionic channels like the sulphate and phosphate anion channels [46, 47] (Fig. **1**). Once inside the RBC, the Cr (VI) is rapidly reduced to unstable intermediates (i.e*.,* short-lived Cr (V) and Cr (IV) species) and ultimately to Cr (III) which becomes bound to haemoglobin and other intracellular proteins [46-50] resulting in increased total chromium levels that remain elevated in the RBC fraction of blood for several weeks, while plasma chromium levels return rapidly to background. Gray & Sterling in 1950 reported that on incubation of RBCs with sodium chromate labelled with chromium-51, the chromium-51 enters the RBCs and after reduction to the cationic trivalent state becomes firmly bound to the globin portion of the haemoglobin molecules within the cell [48]. This tagging eventually became the basis for *in vivo* studies of red cell volume and estimation of red cell life span [51]. Cr binds preferentially to the beta chain of the human haemoglobin and also to non-haemoglobin proteins [52] and glutathione [53]. RBC membranes are however relatively impermeable to the cationic trivalent chromium and when varying amounts of radioactive Cr (III) were added to whole blood *in vitro*, almost all of the radioactivity (94-99%) remained in the plasma with an insignificant count retained in the RBC after saline washing. Similar results were obtained *in vivo* [48].

## CHROMIUM (VI) AND TOXICITY

**Fig. (2):**
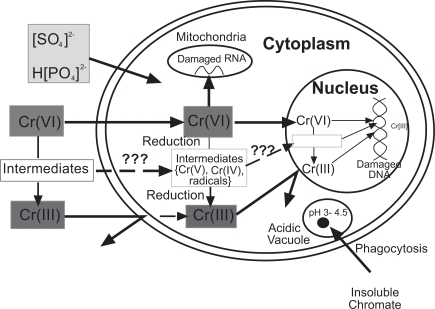
The uptake-reduction model for Cr (VI)-induced carcinogenesis adapted from [57].

Merritt & Brown in 1995 reported that Cr (VI) is the predominant species of chromium released during corrosion of stainless steel and cobalt chrome alloy [38]. Cr (VI) itself does not react with DNA *in vitro* in isolated nuclei but it causes many DNA lesions which include DNA-DNA cross links, DNA-protein cross links and oxidative damage once inside the cell and in the presence of cellular reductants [55, 56]. A reduction-uptake model has been described for Cr (VI)-induced carcinogenesis (Fig. **2**) [56, 57]. Cr (VI) is highly water-soluble and it easily permeates the cell membrane through the physiological anion transport channels. Once inside the cell, reducing enzymes such as NADPH cytochrome c reductase, DT-diaphorase, glutathione reductase and cellular reductants such as cytochrome P_450_, reduced glutathione (GSH) and vitamin C reduce it to reactive intermediates, such as Cr (V) and Cr (IV) [58-61]. During its reduction, reactive oxygen species are produced. These reactive intermediates generated from the *in vivo* reduction of Cr (VI) and reactive oxygen species are thought to be responsible for many of the toxic effects caused by Cr (VI). One of the major intracellular reductants of Cr (VI) is the ubiquitous tripeptide (γ- glutamylcysteinylglycine). Cr (VI) forms a complex with GSH and is subsequently reduced to Cr (V) through a one electron reduction process. The Cr (V) species formed can react *via *the Fenton reaction with hydrogen peroxide to form the DNA damaging hydroxyl radical. Further hydroxyl radical is generated *via *the Fenton mechanism by Cr (IV) formed by the reduction of Cr (V) by cellular reductants like GSH and ascorbate [62]. Several studies supporting the formation of a Cr (V) intermediate in the reaction of Cr (VI) with GSH have been reported [63, 64]. Liu and coworkers [65] reported the synthesis of Cr (IV)-GSH and demonstrated that it can generate hydroxyl radicals in the presence of H_2_O_2._

**Fig. (3):**
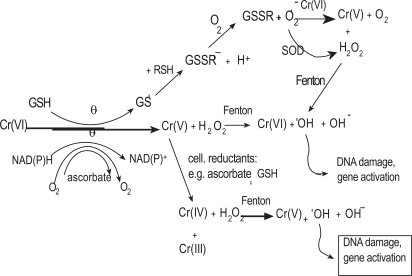
Reduction of Cr (VI) by cellular reductants adapted from [62].

The complex formed by GSH and Cr (VI) and those formed between GSH and the reduced intermediates of Cr (VI) undergo cyclical oxidation and reduction reactions (Fig. **3**) and thus contribute to the redox reactions that mediate the toxicity of the free metal. The serum and tissue concentration of the toxic chromium species may therefore be higher than previously thought when only free chromium was measured by either atomic absorption spectrometry or by inductively coupled plasma-mass spectrometry. It may be necessary to determine the extent of chromium conjugation with GSH in exposed patients and to measure both the free and conjugated chromium ions in serum and tissues in order to have a more realistic idea of the amount of chromium ion released from a M/M implant.

Gunaratnam & Grant in 2004 reported that Cr (VI) induces cell death by apoptosis at low concentrations (260μg/L), and by necrosis at higher concentrations (1300 and 2600μg/L) [66]. The *in vitro* effects of Cr (VI) on the function and viability of osteoblasts have been examined at concentrations that have been earlier reported *in vivo* in patients’ serum. Osteoblasts lose viability after exposure to Cr (VI) at a concentration of 26µg/L for 48hours. Protein, RNA and DNA synthesis are inhibited after exposure to 5.2µg/L for 48 hours. Cr (VI) inhibits collagenase activity and it is thought to inhibit osteoblast collagen fibre synthesis at an intracellular post translational modification stage [67]. Cr (VI) therefore interferes with normal bone turnover and its release from chromium containing alloys would promote implant loosening, both by destroying osteoblasts and by reducing their bone matrix synthesis ability. Cr (VI) has also been shown to cause direct toxicity to the cells of the immune system *in vitro.* It induces oxidative stress and apoptosis in lymphocytes and in mouse macrophages [68-71]. Alterations observed in the cells of the immune system *in vivo* in patients with M/M implants have been attributed to high circulating Cr levels [29]. For example, Hart and colleagues [29] found a 30% decrease in circulating CD8 T-lymphocytes in patients 20 months after implantation, and Savarino and coworkers [72] reported a decrease in CD4 and CD16 positive cells. The consequence of these alterations is unclear.

## EPIDEMIOLOGICAL STUDIES INVOLVING THE INCIDENCE OF CANCER FOLLOWING IMPLANTATION

Cr (VI) has been labeled as a class-1 human carcinogen by the International Agency for Research on Cancer [45] signifying carcinogenesis as a potential long term biological effect in patients with M/M hip replacements [73]. Sunderman [37] suggested that sarcoma at the implantation site of an orthopaedic prosthesis is a complication (though very rare) of metal orthopaedic implants. Onega and coworkers [74] reported an increase in melanoma, which became evident 10 years after arthroplasty. A similar delayed emergence of increased risk of bladder and ureter cancers was also reported. A possible mechanism for these site specific cancers is urinary excretion of metals [28]. However all risks currently remain theoretical as no data have been published to suggest any definite pathological effects. Despite the concern about possible carcinogenicity from chromium and cobalt ion exposure no epidemiological evidence has yet, at this early stage, suggested any associated increase in incidence of any neoplastic disease in arthroplasty related exposure [74, 75]. However, a number of studies have noted chromosomal damage in lymphocytes and bone marrow in patients with metal orthopaedic implants [76-79]. Although such effects do not necessarily mean there is a risk of cancer, it should be borne in mind that similar damage has led to carcinogenesis with other unrelated chemical exposures. It is probable that any pathological effect of metal ion release would take a long time to manifest clinically. It is important to note that the McKee-Farrar prosthesis, a M/M THR prosthesis which was widely used in the 1960s, has not been found to be associated with any pathological effects of chromium or cobalt ion release.

## THE USE OF METAL LEVELS TO MONITOR METAL-ON-METAL IMPLANTS

Several studies have reported a run-in period during the first 2 years after surgery during which metal ion concentrations are significantly elevated. After this run-in period, a steady-state develops in which the ion concentration is constant until the implant begins to fail and the ion concentrations begin to rise again. Ion levels have therefore been suggested as a possible indicator for monitoring implant surface wear and clinical performance of M/M bearings since the predominant source of metal debris in these hips is the articulating M/M surface [80]. Blood and/or urine metal ion analyses have the possibility of providing early confirmation of failure and aid in the timing of a revision operation for a patient with a symptomatic or failed device [34]. This will however become clinically useful after extensive correlative studies establishing the precise relationship between implant performance and ion concentration have been published. Other than the articulating bearing surfaces of the implant other potential sources of metal debris exist and to interpret blood, serum and/or urine levels in patients these sources should be carefully considered. Sources of metal debris like corrosion of fixation screws, non orthopaedic metal devices like heart mechanical valves and environmental sources of metal contamination [34] should all be considered. Care should also be taken to avoid sample contamination during collection since metal ions can be introduced from collection needles. Altered renal function may bias the result and cause an increase in circulating metal levels. This was demonstrated by Brodner and colleagues who reported over 100-fold elevated Co and Cr levels in two patients with renal failure in comparison with individuals with similar implants and normal renal function [81]. The effect of activity level on the extent of metal debris production has been a major controversy. Brodner and colleagues have shown that as the activity level increases, implant wear rate, serum, urine, and blood metal ion levels also increase [81]. They demonstrated this, by reporting the case of a long-distance runner whose Co ion levels increased following a marathon. However, these levels reportedly returned to the baseline after 4 weeks. Contrary to this, Heisel and coworkers reported that the steady-state metal ion level is a function of the amount of debris produced during the initial wear-in period and not dependent on short-term changes in activity level [82]. They reported a constant serum ion level for a given patient with M/M bearing hip prostheses and found no correlation between patient activity and ion levels.

Amstutz and colleagues reported a consensus meeting held in 1996 on the past performance and future directions for M/M implants which discussed extensively the use of ion measurements as a potential means of monitoring M/M wear [6]. It was agreed that sample collection protocol will be prospective and the samples would ideally include serum, red blood cells, urine, and joint fluid. The collection of a synovial biopsy is considered desirable but difficult and probably not practical. Ideal sampling times were determined to be preoperative, immediate postoperative (in hospital); and at 1, 3, 6 months and 1 year, 2 years. Patient demographics, activity and general health will be taken into consideration and kept constant with only the bearing couple; stem fixation type being a variable [6]. MacDonald and coworkers reported that ease of specimen acquisition has made blood and urine the most common samples in trace metal analyses. Good correlation exists between blood and 24-hr urine values but urine samples have a higher likelihood of contamination by the patient. A variation in hydration that may occur throughout the day causes variable metal concentrations in spot urine samples and blood is therefore often the specimen of choice for trace metal analysis. The complexity of matrix components tends to make blood analysis that includes cells (whole blood or erythrocytes) more difficult. Digestion protocols which may theoretically introduce contaminants are necessarily employed to improve the recovery of the metal from whole blood and erythrocytes. The large quantity of iron in the erythrocytes is an additional source of interference [83]. Serum analysis does not have these problems but it gives less characterization of the actual blood level since some metals (particularly chromium) tend to concentrate in erythrocytes. Chromium determination in erythrocytes at a single moment represents a longitudinal result for either the whole lifespan or part of it. This allows the assessment of the stress sustained during the period and it is therefore a superior method compared to determinations in the urine or the plasma which merely represent a snapshot of the situation [49]. To date a comparison of whole blood, serum and erythrocytes on the same specimens in patients with M/M bearings has not been reported [83].

The lack of an established toxicity threshold for the degradation products of cobalt-chrome alloy implants has made monitoring of serum metal levels of little or no use clinically. Occupational safety standards exist, but it is difficult to apply such safety standards to clinical situations of patients with metal implants since they are derived primarily from inhalational exposure to these metal ions rather than a blood-borne route seen in patients with M/M implants. Different routes of exposure would be expected to result in different risk profiles [28, 84].

## MEASUREMENT OF CHROMIUM LEVELS IN BIOLOGICAL FLUIDS

Elemental specific analytic techniques like atomic absorption spectrometry (AAS) have been used for chromium determination in biological materials. Flame atomic absorption spectroscopy (FAAS) was used for quantification of elements in samples in the 1970s. The technology has evolved with the introduction of graphite furnace to atomize the sample. Graphite furnace atomic absorption spectroscopy (GFAAS) allows greater sensitivity of detection and the use of smaller quantities of samples. Inductively coupled plasma-mass spectrometry (ICP-MS) has an exceptional sensitivity combined with high analysis speed. It has a better throughput compared with GFAAS and for most elements, achieves detection limits which are comparable to or better than those of GFAAS. The methods of choice in life sciences must have a high power of detection since the determination of the chromium species in biological samples has to be performed at ultra trace concentration levels. The methods of choice now include GFAAS [9, 13, 25, 40, 81] and ICP-MS [19, 26]. The determination of chromium levels in biological samples is however difficult because of matrix interference and the very low concentrations present in these samples [83].

## CONCLUSIONS

Metal alloy implants are employed in many aspects of modern orthopaedic surgery. Most recently CoCr has been increasingly used in M/M articulating bearings. Elevated Cr levels have been reported to cause DNA and chromosomal damage, reduction in CD8 lymphocyte levels and possibly hypersensitivity reactions (ALVAL). However, to date there has been no significant epidemiological evidence to link any of these changes with an increased incidence in any reported disease. To establish what might be an acceptable level of chromium, guidelines require to be established to standardize measurement of ion concentration and bioavailability in this complex subject.

## Figures and Tables

**Table 1.** Compositional Limits % (m/m) of Elements in the Main Alloys Used in Orthopaedic Prostheses (British Standard Metallic Materials for Surgical Implants http://bsonline.bsi-global.com) [14]

Group	C	Si	Mn	P	S	N	Cr	Co	Mo	Ni	Cu	Fe	Ti	H	O	Al	V	W	Be	Nb	Ta
ISO 5832-1 Wrought stainless steel	0.03	1.0	2.0	0.025	0.01	0.1	17-19	-	2.25-3.5	13-15	0.5	Bal	-	-	-	-	-	-	-	-	-
ISO 5832-2 Unalloyed titanium	0.03	-	-	-	-	0.012	-	-	-	-	-	0.1	Bal	0.0125	0.1	-	-	-	-	-	-
ISO 5832-3 Wrought Ti 6-Al-4 V alloy	0.08	-	-	-	-	0.05	-	-	-	-	-	0.3	Bal	.015	0.2	5.5-6.75	3.5-4.5	-	-	-	-
ISO 5832-4 Co-Cr-Mo casting alloy	0.35	1.0	1.0	-	-	-	26.5-30	Bal	4.5-7.0	1.0	-	1.0	-	-	-	-	-	-	-	-	-
ISO 5832-5 Co-Cr-W-Ni alloy	0.15	1.0	2.0	-	-	-	19-21	Bal	-	9-11	-	3.0	-	-	-	-	-	14-16	-	-	-
ISO 5832-6 Wrought Co-Ni-Cr-Mo alloy	0.025	0.15	0.15	0.015	0.01	-	19-21	Bal	9-10.5	33-37	--	1.0	1.0	-	-	-	-	-	-	-	-
ISO 5832-7 Co-Cr-Ni-Mo-Fe alloy	0.15	1	1-2.5	0.015	0.015	-	18.5-21.5	39-42	6.5-8.0	14-18	-	Bal	0.5-3.5	-	-	-	-	-	0.001	-	-
ISO 5832-8 Wrought Co-Ni-Cr-Mo-T-Fe alloy	0.05	0.5	1.0	-	0.01	-	18-22	Bal	3-4.0	15-25	-	4-6.0	-	-	-	-	-	3-4.0	-	-	-
ISO 5832-9 High nitrogen stainless steel	0.08	0.075	2-4.25	0.025	0.01	0.25-0.5	19.5-22	-	2-3.0	9-11	0.25	Bal	-	-	-	-	-	-	-	0.25-0.8	-
ISO 5832-10 Wrought Ti 5-Al, 2,5 Fe alloy	0.08	-	-	-	-	0.05	-	-		-	-	2-3.0	Bal	0.015	0.2	4.5-5.5	4.5-5.5	-	-	-	-
ISO 5832-11 Wrought Ti 6- Al, 7-Nb alloy	0.08	-	-	-	-	0.05	-			-	-	0.25	Bal	0.009	0.2	5.5-6.5	-	-	-	6.5-7.5	0.5
ISO 5832-12 Wrought Co-Cr-Mo alloy	0.35	1	1	-	-	0.25	26-30	Bal	5-7	1	-	0.75	-	-	-		-	-	-	-	-

Carbon (C), Silicon (Si), Manganese (Mn), Phosphorus (P), Sulfur (S), Nitrogen (N), Chromium (Cr), Cobalt (Co), Molybdenum (Mo), Nickel (Ni), Copper (Cu), Iron (Fe), Titanium (Ti), Hydrogen (H), Oxygen (O), Aluminum (Al), Vanadium (V), Tungsten (W), Beryllium (Be), Niobium (Nb), Tantalum (Ta), Balance (Bal).

**Table 2.** Concentrations of Chromium µg/L in the Biological Samples of Patients Following Ion Release from Different Models of Metal-On-Metal Orthopaedic Implants

Study	Follow Up Years	Model	Serum	Control (Serum)
[37]	2.5	Co-Cr	0.10* ± 0.02	0.1*± 0.04
[38]	> 1	Co-Cr	Plasma RBC 8.31 14.83	Plasma & RBC 0.01
[9]	25	Mckee -Farrar	1.28*	0.14*
	1	Surface replacement	3.86*	0.14*
[40]	2.2	Metasul	1.72* ± 0.33	0.25* ± 0.05
[41]	0.25	Conserve plus	1.88* ± 0.48	0.08* ± 0.04
	0.5		2.02* ± 0.98	
	1		1.80* ± 0.45	
[25]	1.33	Ultima (CoCrMo)	0.99*	0.26*
	1.33	Ultima (CoCrMo) resurfaced	2.77*	0.26*
[30]	Imm postop	metasul	7.78*** ± 2.92	
	0.25-0.5		16.79*** ± 5.9	
	1-1.25		34.75*** ± 7.85	
	3		48.04*** ± 14.08	
	3.5-4		25.62*** ± 19.37	0.21***
[30]	Imm postop	Sikomet	14.27***± 6.46	0.21***
	0.25-0.5		22.19*** ± 10.44	
	1-1.25		30.81*** ± 16.09	
	3		22.24 ***± 9.47	
	3.5-4		36.35*** ± 19.40	
[19]	2	CoCrMo	2.50**	1.09**
[39]	0.5	metasul	0.73* ± 0.76	
	1		1.05* ± 0.76	
	1.5		1.37* ± 090	
[31]	1	Metasul THR	1.7***	
	1	BHR	2.4***	
[29]	0.5-3.5	M/M resurfacing	1.78***	0.28***
[26]	1.28	Conserve Plus	2.88* ± 2.22	1.47* ± 0.37
[27]	6	M/M	3.48*± 2.67	0.92* ± 0.89
[42]	4.5	Metasul	2.1* ± 0.35	0.28* ± 0.04
[13]	5 (retrospective)	Sikomet	1.31* ± 1.37	0.3*± 0.05
	0.4 (Prospective)	Sikomet	1.12* ± 0.54	0.67* ± 0.25
	1.6 (Prospective)	Sikomet	1.30* ± 0.3	0.67* ± 0.25
	0.41(Revision)	Sikomet	0.63*±0.28	Prior to revision 0.79*
	1.28 (Revision)	Sikomet	0.45*±0.148	Prior to revision 0.79*

* Serum (mean values)

** Erythrocyte (median Values)

*** whole blood (mean values).
